# Effective method of measuring the radioactivity of $\left[{\;}^{131}I\right]$‐capsule prior to radioiodine therapy with significant reduction of the radiation exposure to the medical staff

**DOI:** 10.1120/jacmp.v17i4.5942

**Published:** 2016-07-08

**Authors:** Ulf Lützen, Yi Zhao, Marlies Marx, Thea Imme, Isong Assam, Frank‐Andre Siebert, Juraj Culman, Maaz Zuhayra

**Affiliations:** ^1^ Department of Nuclear Medicine Molecular Diagnostic Imaging and Therapy, University Hospital of Schleswig‐Holstein (UKSH) Campus Kiel Germany; ^2^ Clinic of Radiotherapy, Dept. of Medical Physics University Hospital of Schleswig‐Holstein (UKSH) Campus Kiel Karl Germany; ^3^ Institute of Clinical and Experimental Pharmacology University Hospital of Schleswig‐Holstein (UK‐SH) Campus Kiel Germany

**Keywords:** radiation protection, radiation exposure, radioiodine therapy, $\left[{\;}^{131}I\right]$‐capsule measurement

## Abstract

Radiation Protection in Radiology, Nuclear Medicine and Radio Oncology is of the utmost importance. Radioiodine therapy is a frequently used and effective method for the treatment of thyroid disease. Prior to each therapy the radioactivity of the $\left[{\;}^{131}I\right]$‐capsule must be determined to prevent misadministration. This leads to a significant radiation exposure to the staff. We describe an alternative method, allowing a considerable reduction of the radiation exposure. Two $\left[{\;}^{131}I\right]$‐capsules $\left(A_{01}=2818.5;\;A_{02}=73.55.0\;\text{MBq}\right)$ were measured multiple times in their own delivery lead containers — that is to say, $\left[{\;}^{131}I\right]$‐capsules remain inside the containers during the measurements (shielded measurement) using a dose calibrator and a well‐type and a thyroid uptake probe. The results of the shielded measurements were correlated linearly with the $\left[{\;}^{131}I\right]$‐capsules radioactivity to create calibration curves for the used devices. Additional radioactivity measurements of 50 $\left[{\;}^{131}I\right]$‐capsules of different radioactivities were done to validate the shielded measuring method. The personal skin dose rate $\left(H_{P}(0.07)\right)$ was determined using calibrated thermo luminescent dosimeters. The determination coefficients for the calibration curves were $R^{2}>0.9980$ for all devices. The relative uncertainty of the shielded measurement was <6.8%. At a distance of 10 cm from the unshielded capsule the $H_{P}(0.07)$ was 46.18 μSv/(GBq⋅s), and on the surface of the lead container containing the $\left[{\;}^{131}I\right]$‐capsule the $H_{P}(0.07)$ was 2.99 and 0.27 μSv/(GBq⋅s) for the two used container sizes. The calculated reduction of the effective dose by using the shielded measuring method was, depending on the used container size, 74.0% and 97.4%, compared to the measurement of the unshielded $\left[{\;}^{131}I\right]$‐capsule using a dose calibrator. The measured reduction of the effective radiation dose in the practice was 56.6% and 94.9 for size I and size II containers. The shielded $\left[{\;}^{131}I\right]$‐capsule measurement reduces the radiation exposure to the staff significantly and offers the same accuracy of the unshielded measurement in the same amount of time. In order to maintain the consistency of the measuring method, monthly tests have to be done by measuring a $\left[{\;}^{131}I\right]$‐capsule with known radioactivity.

PACS number(s): 93.85.Np, 92.20.Td, 87.50.yk, 87.53.Bn

## I. INTRODUCTION

Radioiodine therapy was first performed in the early forties of the last century^1^, ^2^ and until now, it was a safe and effective method for the treatment of benign and malign diseases of the thyroid gland. In most cases, the radioiodine required for the therapy is administered as a capsule containing $\left[{\;}^{131}I\right]$iodid. In case of benign thyroid diseases, the required radioactivity of the $\left[{\;}^{131}I\right]$‐capsule has to be determined individually for each patient (e.g., by using the Marinelli formula^3^, ^4^) and it depends on the prescribed focal dose, the thyroid volume, $\left[{\;}^{131}I\right]$‐iodine uptake, and the effective half‐life.

According to the German regulations, the $\left[{\;}^{131}I\right]$‐capsule radioactivity must be measured prior to the radioiodine therapy to prevent misadministration.^(5)^ This is usually done by the measurement of the $\left[{\;}^{131}I\right]$‐capsule in a dose calibrator, which might lead to significant exposure to the staff.

A large number of previous studies dealing with the topic of radiation protection mainly focused on general rules of handling radioactive material^6^, ^7^, ^8^, ^9^, ^10^, ^11^, ^12^ and contamination,^(13)^ or provide definite rules and regulations from expert panels (i.e., dose reference values for patients treated or diagnosed using radiation).^10^, ^14^, ^15^, ^16^, ^17^, ^18^

However studies dealing with this particular topic could not often give recommendations how to reduce radiation exposure of the medical staff. The determination of the $\left[{\;}^{131}I\right]$‐capsule radioactivity (e.g., by measuring the local dose rate without taking the $\left[{\;}^{131}I\right]$‐capsule out of the shielding) would result in a considerable lowering of radiation exposure for the medical staff^(19)^ and would be conform with the required principle “as low as reasonably achievable” (ALARA) in terms of radiation protection.^(6)^ In this study, we aim to develop a concrete instruction for lowering radiation hazard during the measurement of the $\left[{\;}^{131}I\right]$‐capsule used for radioiodine therapy. Our study describes a method for the measurement of the $\left[{\;}^{131}I\right]$‐capsule radioactivity while the $\left[{\;}^{131}I\right]$‐capsule remains shielded in its delivery lead container, thus reducing the radiation burden of the staff.

## II. MATERIALS AND METHODS

### A. Measurement of the dose rate Hp(0.07)

**Figure 1:**
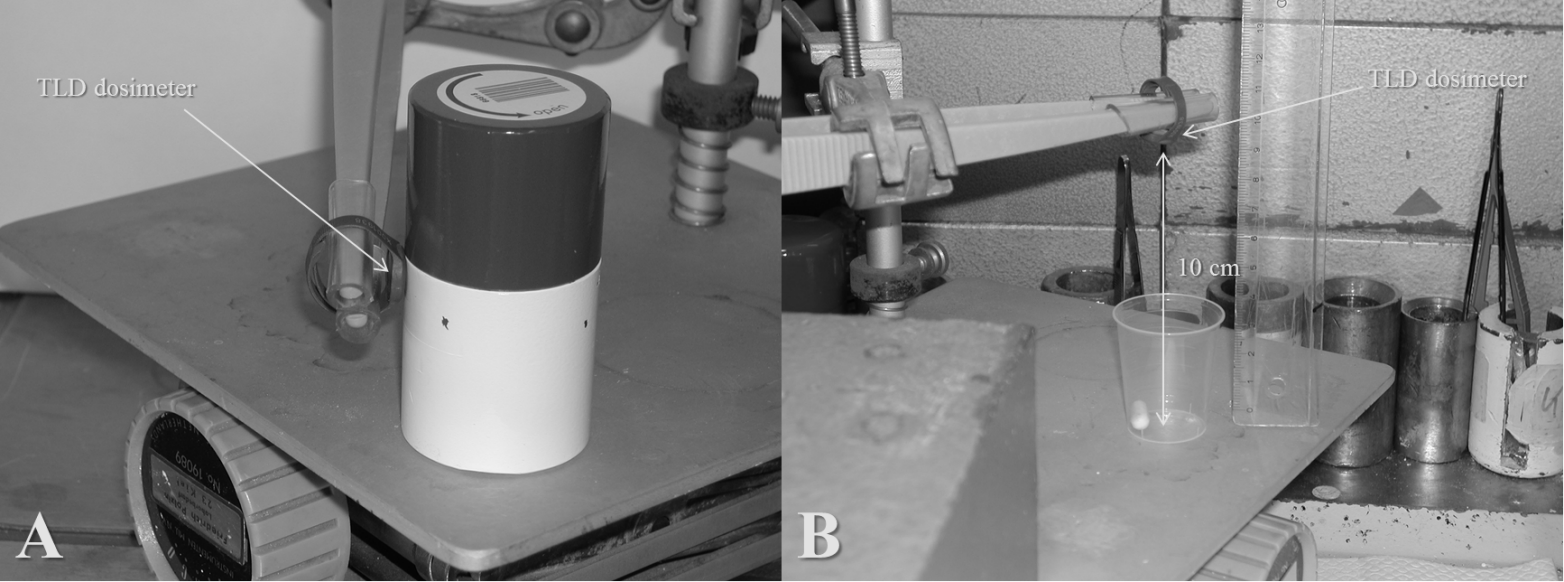
Measurement of the personal skin dose rate $\left(H_{P}(0.07)\right)$ of the $\left[{\;}^{131}I\right]$‐capsule with TLD ring dosimeter: (a) on the side of a size I lead delivery container, and (b) at a distance of ten centimeters without shielding.

The measurements of the personal surface dose equivalent $\left(H_{P}(0.07)\right)$ were performed as shown in Fig. 1, using calibrated official thermo luminescent dosimeters (TLD) (Type X for beta and gamma radiation, GSF‐Forschungszentrum für Umwelt und Gesundheit GmbH, Neuherberg, Germany). The TLDs were placed directly on the surface of the sidewall in the middle of the containers, as shown in Fig. 1(a), as well as at distances of 10 cm and 1 m to the containers. Additional measurements were performed at a 10 cm distance to the unshielded capsules (Fig. 1(b)).

### B. $\left[{\;}^{131}I\right]$‐therapy capsules and measuring devices

**Figure 2:**
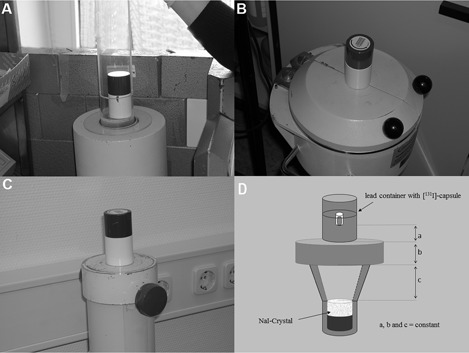
Measurement geometries of shielded $\left[{\;}^{131}I\right]$‐capsule inside the lead container of size I: (a) measuring on the dose calibrator, (b) measuring on the well‐type counter, and (c) measuring on the thyroid uptake probe; (d) measurement geometry demonstrating constant geometric parameters.

Two $\left[{\;}^{131}I\right]$‐capsules with the radioactivities $A_{01}=2818.5\;\text{MBq}$ and $A_{02}=7355.0\;\text{MBq}$ (Mallinckrodt, Dublin, Ireland) were used in the study. The $\left[{\;}^{131}I\right]$‐capsule radioactivities were determined previously using a calibrated dose calibrator (Curiementor 2; PTW, Freiburg, Germany). The radioactivity of the shielded $\left[{\;}^{131}I\right]$‐capsule in the delivery lead container was measured, as demonstrated in Fig. 2, at defined time points according to Table 1 using the dose calibrator, a well‐type counter (5.08×5.08 cm NaI detector with Multi‐Logger LB 5310 data system; Berthold Technologies, Bad Wildbach, Germany) and a thyroid uptake probe (Atomlab 950; Biodex Medical Systems, Baltimore, MD). The usual quality checks for all devices were performed before $\left[{\;}^{131}I\right]$‐capsule measurements using a Cesium‐137 test source (daily and performance check). No additional calibrations and checks were necessary for the implementation of our procedure.

**Table 1:** Measuring time plan showing the measuring time points and actual radioactivities of both used $\left[{\;}^{131}I\right]$‐capsules with the initial activities of $A_{01}=2818.5\;\text{MBq}$ and $A_{02}=7355.0\;\text{MBq}$. At each measurement point the radioactivity was determined first unshielded in the dose calibrator followed by five shielded measurements in five different lead containers with identical specifications for each container size and each device

*Measuring Point*	*Measuring Time Point (Day)*	*Radioactivity of the* $\left[{\;}^{131}I\right]$‐*capsule Measured in the Containers of Size I (MBq)*	*Radioactivity of the* $\left[{\;}^{131}I\right]$‐*capsule Measured in the Containers of Size II (MBq)*
1	3	2147.4	5603.8
2	4	1980.9	5169.2
3	5	1812.3	4729.2
4	6	1649.9	4305.5
5	8	1424.6	3717.6
6	9	1308.7	3414.9
7	11	1078.0	2813.1
8	13	908.2	2370.0
9	16	709.8	1852.1
10	19	543.3	1417.7
11	24	351.9	918.2

### C. Lead containers

**Figure 3:**
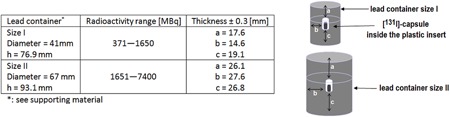
Characteristics and dimensions of size I and size II lead delivery containers.

The $\left[{\;}^{131}I\right]$‐capsules were delivered in lead containers from the same company (Mallinckrodt, Dublin, Ireland). The delivery lead containers are standardized with respect to geometry and material properties (see Appendix A). The $\left[{\;}^{131}I\right]$‐capsule is placed perpendicularly inside a plastic insert of 1 mm thickness, which is fixed on the bottom of a narrow cylindrical bore of the lead container (Fig. 3). The containers are made of 99% lead and their specifications are summarized in Fig. 3. The maximum dimension tolerance is 0.3 mm. $\left[{\;}^{131}I\right]$‐capsules with radioactivities ranging from 370–1650 MBq and from 1651–7400 MBq were delivered inside containers of size I and II, respectively. To cover the calibration ranges of 370–1650 MBq and 1651–7400 MBq for both container sizes, the measurements of the two $\left[{\;}^{131}I\right]$‐capsules ($A_{01}=2818.5\;\text{MBq}$ and $A_{02}=7355.0\;\text{MBq}$) were distributed within a period of 24 days over 11 measurement time points, as demonstrated in Table 1. At every measuring time point, the $\left[{\;}^{131}I\right]$‐capsule radioactivity was measured first unshielded in the dose calibrator and then shielded in five different lead containers with identical specifications of the same supplier, using the three devices as shown in Fig. 2. Because the containers of size II were too large to fit into the well chamber of the dose calibrator, they were excluded from the study and the total number of the measurements was only n=25×11=275 (instead of 30×11=330). The measuring time for all devices was as long as it was necessary to reach a statistical accuracy of 1% (10 to 75 s).

To verify the container geometry and material properties, a $\left[{\;}^{131}I\right]$‐standard (A=100 MBq, Physikalisch‐Technische Bundesanstalt PTB, Braunschweig, Germany) was measured successively inside of five different lead containers with identical specifications of each size using the three devices (five measurements for each device and container size). The relative error of the $\left[{\;}^{131}I\right]$‐standard was stated as 1.0% by the supplier.

### D. Regression lines and linear calibration factors

**Figure 4:**
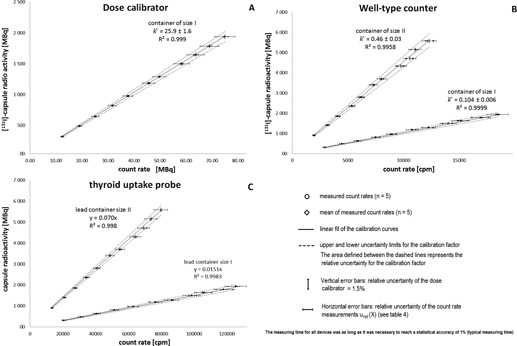
Fit of the linear calibration curves for the shielded measurement method of the $\left[{\;}^{131}I\right]$‐capsule radioactivity ($\left[{\;}^{131}I\right]$‐capsule in delivery lead container) indicating the calibration factor values and the determination coefficients: (a) dose calibrator, (b) well‐type counter, and (c) thyroid uptake probe. Y‐axis: $\left[{\;}^{131}I\right]$‐capsule radioactivity measured regularly unshielded in the dose calibrator; x‐axis: $\left[{\;}^{131}I\right]$‐capsule radioactivity measured shielded by leaving the $\left[{\;}^{131}I\right]$‐capsule inside of the delivery lead containers.

To create linear calibration curves for every device and container size, the arithmetic mean of the count rates obtained from the repeated measurements of the $\left[{\;}^{131}I\right]$‐capsule inside of five different lead containers with identical specifications (n=5) at every measuring time point (Table 1) was correlated with the radioactivity of the unshielded capsules measured before in the dose calibrator. As a result, in total for all devices, five linear calibration curves were obtained, each fitted to 11 data points (Fig. 4).

Giving that $y_{i}$ (the radioactivity of the unshielded $\left[{\;}^{131}I\right]$‐capsule measured with a dose calibrator) and $x_{i}$ (the count rate of the shielded $\left[{\;}^{131}I\right]$‐capsule measured with the dose calibrator, well‐type counter or the thyroid uptake probe) were correlated linearly, the relationship can be mathematically described as an equation of a straight line with the slope $\hat{k}$:

(1) $$y_{i}=\hat{k}\cdot x_{i}$$

Because both correlated values $x_{i}$ and $y_{i}$ are affected by measurement errors, we calculated the calibration factor $\hat{k}$ using the compensation of a straight line for both coordinates Y and X, which is known as regular compensation or regression line for both coordinates, as described by Matus.^(20)^ We used this compensation because it also guarantees the independence of the calibration factor from the units used for X and Y.^(21)^

(2) $$\hat{k}=sign\cdot [\sum_{i}^{n}(x_{i}-\bar{x})(y_{i}-\bar{y})]\cdot \sqrt{\frac{\sum_{i}^{n}{\left(y_{i}-\bar{y}\right)}^{2}}{\sum_{i}^{n}{\left(x_{i}-\bar{x}\right)}^{2}}}$$

where $\hat{k}$ is the slope of the regression line, $y_{i}$ represents the radioactivities of the unshielded $\left[{\;}^{131}I\right]$‐capsule measured in the dose calibrator, $x_{i}$ represents the count rates of the shielded capsule, and $\bar{x}$ and $\bar{y}$ are the mean of $x_{i}$ and $y_{i}$.

### E. Uncertainties of the measurements

The uncertainty, u(device), of the dose calibrator, the well‐type counter and the thyroid uptake probe, was determined by using a calibrated $\left[{\;}^{131}I\right]$‐standard (A=100 MBq, Physikalisch‐Technische Bundesanstalt PTB). The relative uncertainty of the activity of the $\left[{\;}^{131}I\right]$‐standard was stated as 1.0% by the supplier. The measurement of the device precisions was performed using the primary test procedure according to the “German Industrial Standard” (DIN) and the “Guide to the Expression of Uncertainty in Measurement” (GUM).^22^, ^23^ (For more details see Appendix B.)

The maximum uncertainty that may result during the shielded measurement of the $\left[{\;}^{131}I\right]$‐capsule inside the lead containers, u(lead), was calculated from the indicated maximum dimensional tolerance of the lead containers stated by the supplier of ±0.3 mm using the attenuation of the $\left[{\;}^{131}I\right]$‐gamma radiation for lead given in the literature (see Fig. 15.28 k in Vogt and Schultz^(24)^) when varying the lead thicknesses of the containers of 19.1 mm and 26.8 mm, respectively, within the tolerance of ±0.3 mm. u(lead) was additionally confirmed statically by measuring the $\left[{\;}^{131}I\right]$‐standard successively inside of five different lead containers with identical specifications of each size using the three devices and estimating the standard deviation (SD).

### F. Validation of the measurement method

**Figure 5:**
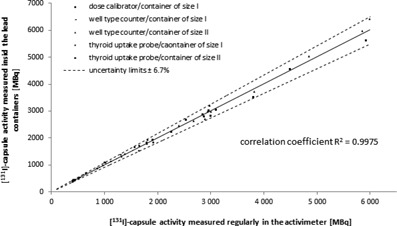
Correlation between the $\left[{\;}^{131}I\right]$‐capsule activity values obtained from the unshielded measurement in a dose calibrator and the shielded measurement of the activities using the calibrated devices. The area defined between the dashed lines represents the relative uncertainty limit of the shielded measuring method (±6.7%).

After establishing the calibration curves for all devices, we validated our method by measuring 50 additional $\left[{\;}^{131}I\right]$‐capsules (10 per lead container size and device, sample size n=10) that were delivered later from the same supplier. The capsules were measured first inside the delivered original containers using the calibrated devices followed by the unshielded measurements with the dose calibrator. The values from both methods were then correlated and the common correlation factor for all devices and container sizes was determined (Fig. 5).

In order to demonstrate the radiation dose reduction of our procedure, we measured the hand $\left(H_{P}(0.07)\right)$ and body doses $\left(H_{P}(10)\right)$ in the practice for both methods using official finger ring thermoluminescent dosimeters and body film dosimeters. For this reason, two I‐131‐capsules with start radioactivities of 6.000 and 1.650 GBq for the container of size I and II were repeatedly measured both with our shielded method and with the conventional unshielded method. For comparability purposes, the total measured radioactivity for each container size was the same for both measuring methods. For the container of size I 81.5 GBq (n=91 measurements, mean=0.9 GBq) were measured, and for the container of size II the total measured radioactivity was 218.1 GBq (n=60 measurements, mean=3.8 GBq). Five persons were involved in the measurement to minimize the probability of systematic errors and to reduce the radiation dose of the individuals. The five persons were wearing alternately different official finger ring and body film dosimeters, depending on the measuring method and the used container size (n=4 for each type of dosimeters). The dosimeters were then officially evaluated.

## III. RESULTS

Table 2 summarizes the results from the $H_{P}(0.07)$ measurements at different distances to the surface of the shielding containers containing the $\left[{\;}^{131}I\right]$‐capsule and to the unshielded capsules.

The determination of the relative uncertainties for the dose calibrator, the well‐type counter, and the thyroid uptake probe, by using the calibrated $\left[{\;}^{131}I\right]$‐standard, results in u(device)=1.5% for all devices.

The results of the measurements of the $\left[{\;}^{131}I\right]$‐standard in five different lead containers and the statistically estimated ${\text{ur}}_{\text{el}}$(lead) are illustrated in Table 3. The highest value for $u_{\text{rel}}$(lead) was calculated for the container of size 1 and the measurement with the well‐type counter $u_{\text{rel}}(\text{lead})=4.6\%$. Using the attenuation of $\left[{\;}^{131}I\right]$‐gamma radiation from the literature,^(24)^ we estimated a relative uncertainty $u_{\text{rel}}(\text{lead})=\pm 5\%$ when varying the lead thicknesses of the container of 19.1 mm and 26.8 mm, respectively, within the tolerance of ±0.3 mm.

**Table 2:** Comparison of the Hp(0.07) in [(μSv/GBq⋅s)] measured in 10 cm to the unshielded capsule as well as on the surface of the lead containers and in 10 and 100 cm distances to the surface

*Distance*	*Capsule in Container of Size I* [(μSv/GBq⋅s)]	*Capsule in Container of Size II* [(μSv/GBq⋅s)]	*Capsule Without Shielding* [(μSv/GBq⋅s)]
surface	2.99	0.27	‐
10 cm	0.99	0.09	46.18
100 cm	0.010	0.001	‐

**Table 3:** Results of the shielded measurements of the $\left[{\;}^{131}I\right]$‐standard (A=100 MBq,u=±1%) inside of the five different lead containers with identical specifications. The statically estimated maximum relative uncertainty was found for the well‐type counter and container size I (4.6%)

*Device*	*Container*			*Count Rate (cps)*			*Mean (m)*	*SD*	$\mu_{rel}(lead)=\sqrt{{\left(\frac{SD}{m}\right)}^{2}+\mu_{rel}^{2}(I-13\;1-standard)}$ ^b^
Dose calibrator	size I	4.2^a^	3.9^a^	3.8^a^	3.9^a^	4.1^a^	4.0	0.2	4.2%
Well‐type counter	size I size II	850 225	922 235	883 233	925 227	957 210	929 228	41.5 9.8	4.6% 4.4%
Thyroid uptake probe	size I size II	6615 1500	6502 1380	6110 1411	6435 1360	6825 1467	6528 1436	262.3 58.8	4.1% 4.2%

a ^a^ Values in MBq

b ^b^ Calculated from the SD and the relative uncertainty of the $\left[{\;}^{131}I\right]$‐standard stated as 1.0% by the supplier using the primary test method.^20^, ^27^

Table 4 summarizes the calculated relative uncertainties for the $\left[{\;}^{131}I\right]$‐capsule radioactivity in the dose calibrator $u_{\text{rel}}$(Y), for the count rate measurements $u_{\text{rel}}$(X), the relative uncertainties of the calibration factors $u_{\text{rel}}(\hat{k})$ and the total combined relative uncertainty of the method $u_{\text{rel}}$(A). These results are also included in Fig. 3 demonstrating the linear calibration curves and the calibration factors for the respective devices, as well as the determination coefficients for the linear fits and the uncertainty of the calibration factors indicated as the area between the dashed lines.

Figure 5 shows the results of the validation measurements as a correlation between regularly measured $\left[{\;}^{131}I\right]$‐capsule radioactivities with the dose calibrator and the measured radioactivities of the shielded $\left[{\;}^{131}I\right]$‐capsule in the delivery lead container using the calibrated devices. As shown in Fig. 5, the measured values from both methods are linearly correlated $\left(R^{2}=0.9975\right)$ and located within the range which is defined by the total uncertainty of the measuring method, $\mu_{\text{rel}}(A)=\pm 6.7\%$.

Table 5 shows the results of the determination of the hand doses caused during the measurement of a total ^131^I‐capsule radioactivity of 81.5 GBq for container of size I and 218.1 GBq for container of size II by applying both measuring procedures. The reduction of the effective dose was 56.6% when the container of size I was used and 94.9% by using the container of size II. The whole‐body doses were under the detection limit of the body film dosimeters for both methods (<200μ Sv).

**Table 4:** Summary of calculated relative uncertainties

	*Dose Calibrator*	*Well‐Type Counter*		*Thyroid Uptake Probe*	
	*Container of size I*	*Container of size I*	*Container of size II*	*Container of size I*	*Container of size II*
$u_{\text{rel}}(X)=\sqrt{\mu_{\text{rel}}^{2}(\text{device})+\mu_{\text{rel}}^{2}(\text{lead})+\mu_{\text{rel}}^{2}(\text{sta}.)}$	5.2%	5.2%	5.2%	5.2%	5.2%
$u_{\text{rel}}(\hat{k})=\frac{\sqrt{\sum \frac{{\hat{k}}^{2}\cdot {\left(x_{i}-\bar{x}\right)}^{2}\cdot u^{2}(x_{i})}{\sum_{i}^{n}{\left(x_{i}-\bar{x}\right)}^{2}}+\sum \frac{{\hat{k}}^{2}\cdot {\left(y_{i}-\bar{y}\right)}^{2}\cdot u^{2}(y_{i})}{\sum_{i}^{n}{\left(y_{i}-\bar{y}\right)}^{2}}}}{\hat{k}}$	4.2%	4.1%	4.2%	4.1%	4.1%
$u_{\text{rel}}(A)=\sqrt{\mu_{\text{rel}}^{2}(\hat{k})+\mu_{\text{rel}}^{2}(\text{device})+\mu_{\text{rel}}^{2}(\text{lead})+\mu_{\text{rel}}^{2}(\text{sta}.)}$	6.7%	6.7%	6.7%	6.6%	6.7%

$[\mu_{\text{rel}}(x)]=$ relative uncertainty of the count rate measurements by leaving the capsule inside the lead container; $\mu_{\text{rel}}(k)=$ relative uncertainty of the calibration factor; μ_rel_(A) total combined relative uncertainty of the measurement method; $\mu_{\text{rel}}(\text{device})=1.5\%$, relative uncertainty of the devices used for the shielded $\left[{\;}^{131}I\right]$‐capsule measurement estimated using the $\left[{\;}^{131}I\right]$‐standard; $u_{\text{rel}}(\text{lead})=\pm 5\%$, the maximum relative uncertainty calculated from the manufacturer specifications indicating a maximum tolerance for the dimensions of the lead containers of $\pm 0.3\;\text{mm};\;u_{\text{rel}}(\text{sta}.)=\pm 1\%$, statistical relative error.

**Table 5:** Results of the measurement of the radiation dose caused to the hands and body by the conventional and by our shielded measurement method

*Used Container*	*Measuring Method*	*Sum of Measured I‐131‐activity (GBq)*	*Measured Hand Dose (μSv)* ^a^	*Body Dose (μSv)* ^b^	*Calculated Body Dose (μSv)* ^c^	*Dose Reduction by Our Method*
container size I	our method	81.5	3000	below detection	61.1	56.6%
	conventional method	81.5	21007	limit below detection limit	‐	
container size II	our method	217.1	950	below detection	16.4	94.9%
	conventional method	217.1	511 12	limit below detection limit	‐	

a ^a^ Measured with official finger ring thermo luminescent dosimeters.

b ^b^ Measured with official film dosimeters (detection limit <200μ Sv).

c ^c^ The values were calculated from the local dose rate in a distance of 1 m to the shielded capsule from Table 2 and a maximum measuring time of 75 s.

## IV. DISCUSSION

Exactly defined geometric conditions of the measurements using the well‐type counter and the thyroid uptake probe could be achieved by marking the contours of the transport lead containers on the lead cover of both devices for the exact position of the transport lead containers (Figs. 4(b) and (c)). The exact geometry is also given during the measurement of the lead container in the dose calibrator as this was performed in the same way as the unshielded measurement by putting the lead container inside of the measuring chamber of the dose calibrator (Fig. 4(a)). As demonstrated in Fig. 4(d), the distance between the $\left[{\;}^{131}I\right]$‐capsule and the NaI‐detector, as well as the lead thickness between $\left[{\;}^{131}I\right]$‐capsule and detector, are for every lead container size constant. It is, therefore, guaranteed that the radiation intensity attenuation is always the same and therefore a linear correlation between the radioactivity of the $\left[{\;}^{131}I\right]$‐capsule inside the lead container and the measuring signal can be assumed.

The more crucial issue affecting the uncertainty of the measurement is the dimension tolerance of the lead container. As in our case, the $\left[{\;}^{131}I\right]$‐capsule supplier is the same for both the $\left[{\;}^{131}I\right]$‐capsule and the standardized container, we were able to calculate a maximum relative uncertainty of $u_{\text{rel}}(\text{lead})=\pm 5\%$ from the stated maximum tolerance of ±0.3 mm. This value fits to the statistically estimated maximum relative uncertainty by measuring the $\left[{\;}^{131}I\right]$‐radioactivity standard successively inside of five different, but identical, lead containers of each size using the three devices of ±4.6% (Table 3).

Considering all uncertainties, including the container uncertainty, the uncertainty of the calibration factor and the uncertainty of the measuring devices, our study revealed a sufficient final total relative uncertainty of <6.7%. This value is clearly inside the acceptance limits for radioactivity measurements for radiopharmaceuticals (e.g., in the European Pharmaceutical guidelines of ±10%).^(25)^

The person measuring the $\left[{\;}^{131}I\right]$‐capsule radioactivity by the standard method (unshielded capsule) needs to carry the $\left[{\;}^{131}I\right]$‐capsule in the lead container for approximately the same time as also required for measuring with our alternative method. Consequently, he will receive with both methods the same radiation dose for both the hands and whole body.

However, the radiation exposure during the measurement time on the devices is different between both methods. Because the unshielded measurement of the $\left[{\;}^{131}I\right]$‐capsule with the dose calibrator usually takes place in a well‐shielded dose calibrator, primarily the hands are exposed to radiation; the radiation dose to the rest of the body is considered to be negligible. For the estimation of the radiation exposure to the hands, we used a mean of 5 s for completing the measurement procedure (taking out the [^131I^]‐capsule from the lead container, placing the unshielded $\left[{\;}^{131}I\right]$‐capsule in the measuring chamber of the dose calibrator, and replacing the $\left[{\;}^{131}I\right]$‐capsule back in the lead container after the measurement). The average distance from the unshielded $\left[{\;}^{131}I\right]$‐capsule to the hands during the measurement procedure was estimated to be in average 10 cm (use of tweezers).

The measured $H_{P}(0.07)$ at a distance of 10 cm from the unshielded $\left[{\;}^{131}I\right]$‐capsule is 46.18 μSv/(GBq·s) (Table 2). The corresponding estimated radiation dose at 10 cm in 5 s (average time needed to perform the unshielded $\left[{\;}^{131}I\right]$‐capsule measurement using a dose calibrator) is 230.9 μSv/GBq. Applying the tissue weighting factor for the skin, $w_{T}=0.01$,^(26)^ this value corresponds to an effective dose of $H_{\text{eff}}=2.309\;\mu \text{Sv}/\text{GBq}$.

Using our measurement technique, the shielding containers need not be opened. Assuming that the performing person has a distance of 1 m during the measurement to the shielded $\left[{\;}^{131}I\right]$‐capsule and the duration of the correct placement of the lead container inside the marks on the devices is at the most 60 s, the expected effective radiation dose to the body can be easily estimated using the determined $H_{P}(0.07)$ in 1 m distance to the shielded [_131_I]‐capsule (Table 2). We calculated a maximum effective dose of 0.6 μSv/GBq and 0.06 μSv/GBq for container size I and II, respectively.

Based on these data, the theoretically calculated reduction of the effective radiation dose by using our method is 74.0% for the container of size I and 97.4% for the container of size II. However, the determination of the effective dose in the practice revealed a reduction of the effective dose of 56.6% for the container of size I and 94.9% for the container of size II. The clearly lower dose reduction (56.6%) by using the container of size I compared to the theoretically calculated value of 74% is primarily due to the hand dose caused while carrying the container. As shown in Table 2, the measured dose on the container surface is for the container of size I is 2.99 μSv/GBq·s. This value is in fact more than eleven times higher than the value measured for the container of size II (0.27 μSv/GBq·s). Therefore, its contribution to the total hand dose of the conventional unshielded measurement is higher for the container of size I than corresponding dose for the container of size II.

On the other hand the measured whole body doses for both methods were undetectable below the detection limit of the official personal film dosimeter and thus practically negligible.

Because of the significant dose reduction to the staff and due to the simple practicability, our shielded measuring method has been established for the routine measurement of the $\left[{\;}^{131}I\right]$‐capsule in our facility. Our experience has shown a very good reliability without any difficulties.

## V. CONCLUSIONS

The shielded measurement of the $\left[{\;}^{131}I\right]$‐capsule inside of the lead delivery container leads in the praxis to significant reduction of the radiation exposure to the hand of the personnel (93.48%) and enables to measure and verify the manufacturer's information of the delivered radioactivity with high precision. The measuring times are comparable with those of the standard unshielded measuring method using a dose calibrator. However, it must be ensured that only standardized shielding container of the same geometric and material properties with known tolerances of dimensions can be used. In order to maintain the consistency of the measuring method, monthly tests have to be done by measuring a $\left[{\;}^{131}I\right]$‐capsule with known radioactivity. Furthermore, if at any time, discrepancies were found between the measured and declared radioactivity in the documents of the $\left[{\;}^{131}I\right]$‐capsule deliverer, the method has also to be checked by additionally measurement of the unshielded capsule in the chamber of the dose calibrator. In this way, eventually unannounced changes of container specifications by the manufacturer would be detected timely.

## COPYRIGHT

This work is licensed under a Creative Commons Attribution 3.0 Unported License.

## APPENDICES

### Appendix A: Lead Container Dimensions 1

### Appendix B: Lead Container Dimensions 2
